# Dental implant care and trouble among dependent patients based on the questionnaire survey among Japanese dental practitioners

**DOI:** 10.1186/s12903-020-01279-0

**Published:** 2020-11-25

**Authors:** Yuji Sato, Shigeto Koyama, Chikahiro Ohkubo, Shin Ogura, Ryutaro Kamijo, Soh Sato, Jun Aida, Yuichi Izumi, Mihoko Atsumi, Akio Isobe, Shunsuke Baba, Noriharu Ikumi, Fumihiko Watanabe

**Affiliations:** 1 Department of Geriatric Dentistry, Showa University School of Dentistry, 2-1-1, Kitasenzoku, Ohta-ku, Tokyo 145-8515 Japan; 2 Maxillofacial Prosthetics Clinic, Tohoku University Hospital, 1-1, Seiryomachi Aoba-ku, Sendai-shi, Miyagi 980-8574 Japan; 3 Department of Removable Prosthodontics, Tsurumi University School of Dental Medicine, 2-1-3, Tsurumi, Tsurumi-ku, Yokohama-shi, Kanagawa 230-8501 Japan; 4 Division of Oral Implant, The Nippon Dental University Hospital Tokyo, 2-3-16, Fujimi, Chiyoda-ku, Tokyo 102-8158 Japan; 5 Department of Biochemistry, Showa University School of Dentistry, 1-5-8, Hatanodai, Shinagawa-ku, Tokyo 142-8555 Japan; 6 Department of Periodontology, The Nippon Dental University School of Life Dentistry at Niigata, 1-8, Hamauracho, Chuo-ku, Niigata-Shi, Niigata 951-8580 Japan; 7 Department of International and Community Oral Health, Tohoku University Graduate School of Dentistry, 4-1, Seiryomachi Aoba-ku, Sendai-shi, Miyagi 980-8575 Japan; 8 Department of Periodontology, Tokyo Medical and Dental University Graduate School of Medical and Dental Sciences, 1-5-45, Yushima, Bunkyo-ku, Tokyo 113-8510 Japan; 9 Department of Oral Interdisciplinary Medicine, Kanagawa Dental University Graduate School of Dentistry, 82, Inaokacho, Yokosuka-shi, Kanagawa 238-8580 Japan; 10 Department of Oral Implantology, Osaka Dental University, 1-5-17, Otemae, Chuo-ku, Osaka-shi, 540-0008 Japan; 11 Medical Corporation Ishikura Dental Clinic, 457-3, Iizukamachi, Takasaki, Gunma 370-0069 Japan; 12 Department of Crown and Bridge Prosthodontics, The Nippon Dental University School of Life Dentistry at Niigata, 1-8, Hamauracho, Chuo-ku, Niigata-Shi, Niigata 951-8580 Japan

**Keywords:** Elderly people, Implant, Visiting dental treatment, Care, Problems

## Abstract

**Background:**

Self-care and professional care of implants may prove difficult for elderly people who require nursing care. However, the actual state of care and problems remains unknown. In this study, we investigated the actual state of implant problems in elderly people living in their own home or in a nursing home who received visiting dental treatment.

**Methods:**

We mailed questionnaire survey forms to 2339 representatives or specialists who were members of the Japanese Society of Oral Implantology, the Japanese Society of Gerodontology or the Japan Prosthodontic Society. We narrowed down the respondents to those who provided visiting dental treatment, and analyzed the actual state of implants observed during visiting dental treatment (type, care, problems, countermeasures, etc.).

**Results:**

Of the 924 dentists who responded to the questionnaire survey, 291 (22%) provided visiting dental treatment. While the majority of implant types encountered in the previous 12 months were root-form implants, there were still a certain number of blade and subperiosteal implants. Daily implant care involved mostly cleaning with a toothbrush + auxiliary tools. The most frequent implant problems encountered in the past were difficulty in cleaning and peri-implantitis. Medication and antiphlogistic treatment were most frequently adopted as countermeasures to implant problems, followed by observation. When we classified the results into those for the dentists who provided implant treatment and those for the dentists who did not, we found that many of the dentists who did not provide implant treatment opted for observation or medication, while those who provided implant treatment also implemented removal of superstructure, retightening of screws, repair and so forth.

**Conclusions:**

We found that many of the implant troubles encountered by dentists who provided visiting dental care were difficulty in cleaning or peri-implantitis, and that the actions taken against these troubles varied depending on the experience of the dentist performing the implant treatment. Our study also revealed that dentists who provide visiting dental care need to acquire knowledge and skills of implant treatment, to have actions prepared in case they encounter such cases, or to closely coordinate with dentists who specialize in implants.

## Background

According to a report by the Cabinet Office, the proportion of elderly people in Japan has been constantly increasing since 1950, reaching 28.1% (35.58 million people) in 2019, and is expected to keep increasing until 2065 [1]. Furthermore, the number of people who require nursing care now exceeds 6.68 million, which corresponds to approximately 20% [2], and is also expected to increase in the future. Since it is difficult for elderly people who require nursing care to visit clinics and they do not receive sufficient dental or oral care, the condition of their oral cavity is highly likely to be worse than that of outpatients, and they are likely to have various troubles. Meanwhile, the Survey of Dental Diseases in Fiscal 2016 [3], which is a survey conducted every 6 years, showed that 4.6% of people aged 65 to 69 years old use implants, while elderly people aged 80 years and older who do so account for less than 3.0%. Since oral implants have also become a popular method of prosthetic treatment for missing teeth, the number of elderly people who require nursing care and who also use implants is expected to increase, and accordingly, the number of implant troubles is expected to increase. However, elderly people who are admitted to nursing care facilities are not included in this survey, which does not grasp the oral cavity conditions of patients with implants who have difficulty visiting clinics, or the state of provision of visiting dental care. Furthermore, the dentist in charge of the patient is likely to change when a patient with an implant starts requiring nursing care, as the form of care changes from outpatient to visiting care. Therefore, media such as a standardized card that records the types of implant body and abutment as well as the fixing method for prosthetic devices (implant card) are likely to be useful when it is necessary to repair or modify prosthetic devices for implants.

In our previous study, we surveyed 2339 representatives or specialists of the Japanese Society of Oral Implantology, Japanese Society of Gerodontology, and Japan Prosthodontic Society to assess whether implant treatment was provided, whether visiting dental care was provided, trends in implants and patients after treatment, actual states of implants in visiting dental care, and the state of utilization and awareness of implant cards, and obtained responses from 924 [4]. We found that at least 30% of the dentists had patients who had received implant treatment and who were later admitted to a hospital or required home care. We also found that 22% of the dentists had been asked about implants by their patients. The rate of dentists who continued providing care through visits was approximately 80%. However, 40% of the dentists did not grasp the trends of the patients after implant treatment. Approximately 3% of the patients receiving visiting dental treatment had implants (mainly confirmed by visual examination). More than 50% of the dentists who provided implant treatment did not use implant cards, and even when the cards were used, they lacked consistency. It is necessary to expand the provision of continuous care after implant treatment, and we consider that the popularization of cards under a unified standard is essential for achieving this.

In this study, we narrowed down the survey respondents to dentists who provided visiting dental treatment and we analyzed the data on the implant care and problems encountered as well as the countermeasures in order to elucidate the actual state of implants in elderly people requiring nursing care.

## Methods

The survey was conducted as a questionnaire for a period of three months from August to October 2015, with the survey respondents’ names entered. The questionnaire forms were distributed and collected in mail [4]. Table 1 shows the questions that were asked in the questionnaire.

**Table 1:** Survey questions

1. Do you offer implant treatment?	
2. Do you give a “card/pocket notebook” to patients for whom implant treatment has been completed? 3. Among the patients who received implant treatment at your clinic, are there any patients who were admitted to the hospital or became bedridden at home?	
4. Have you been consulted by your implant patients or their families about oral health management when the patients were admitted to the hospital or became bedridden?	
5. If you are informed by one of your implant patients that s/he cannot visit your clinic due to becoming bedridden, how do you address this?	
6. Please provide the number of institutions and patients by the category of institutions you visit for home-visit dental care	
Number of institutions	
Total number of patients who receive your home-visit dental care	
Of the above patients, the total number of patients who are unable to perform oral self-care	
Total number of patients who have implants among those who receive your home-visit dental care	
Of the above patients who have implants, the total number of patients who are unable to perform oral self-care	
7. How do you identify the presence of implants in patients receiving your home-visit dental care?	
8. Would it be helpful if institutionalized or homebound older adults have an implant card/pocket notebook (something like the Prescription Pocket Notebook) or treatment history/information?	

Questionnaire forms were distributed to 2339 representatives or specialists who were members of the Japanese Society of Oral Implantology, the Japanese Society of Gerodontology or the Japan Prosthodontic Society, and we received 924 completed (40% collection rate). We then narrowed down the respondents to those who provided visiting dental treatment and analyzed the data for the following three matters.

1. Actual state of implants and daily implant care in patients receiving visiting treatment
  Types of implants encountered in the previous 12 months
  Types of daily implant care implemented in the previous 12 months
2. Actual state of implant problems
  Types and number of cases of implant problems encountered in the past
3. Actual state of countermeasures for implant problems
  Types and number of cases of countermeasures for implant problems

We used the χ^2^ test to analyze the relationship between the required items.

This study was conducted with the approval of the Ethics Committee of the Japanese Society of Oral Implantology (No.: 2015-1).

## Results

Of the 924 dentists who responded to the survey, 291 dentists (22%) provided visiting dental treatment. Among them, 206 dentists (71%) provided implant treatment. The number of dentists who encountered patients with implants during visiting dental care in the past year was 96 (54 provided implant treatments, 32 did not provide treatments), and the total number of implant patients was 360, which corresponded to 3% of the total number of patients (12,356) [4].

**Fig. 1:**
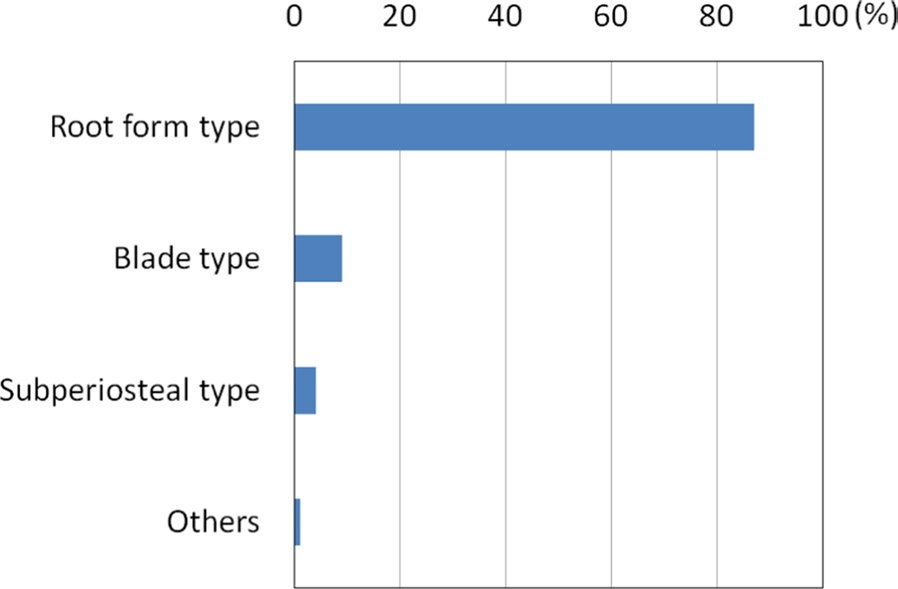
Types of implants encountered in the previous 12 months. While the majority of implant types encountered in the previous 12 months were root-form implants, there were still a certain number of blade and subperiosteal implants

**Fig. 2:**
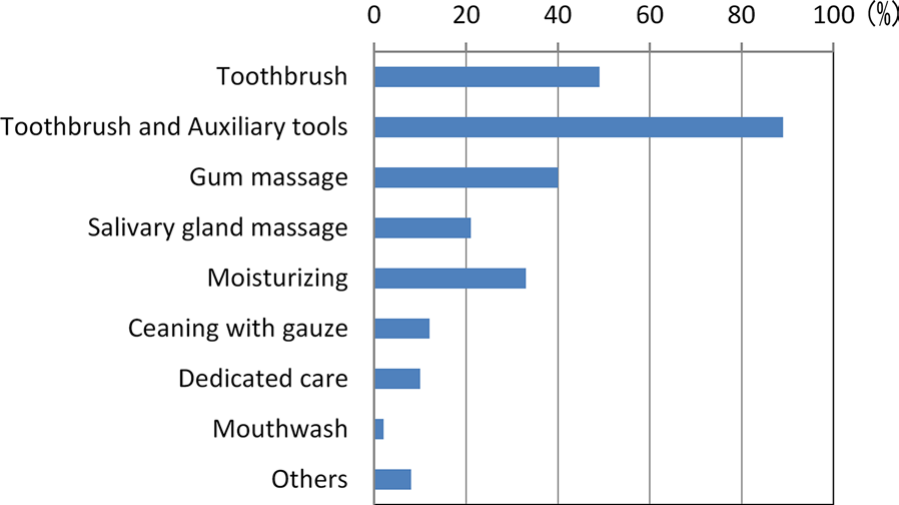
Daily implant care implemented in the previous 12 months. Toothbrush + auxiliary tools (such as interdental brushes) accounted for the majority of cases

**Fig. 3:**
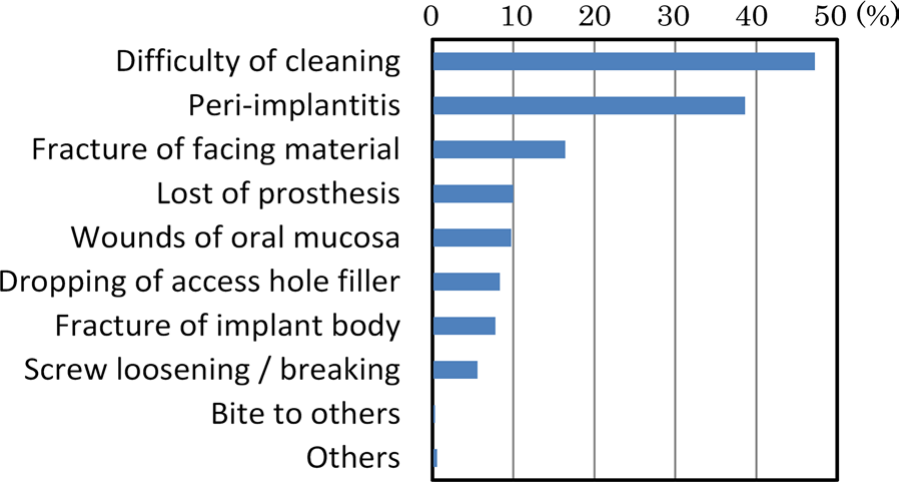
Implant problems encountered in the past. Most frequent problems were difficulty in cleaning and peri-implantitis

**Fig. 4:**
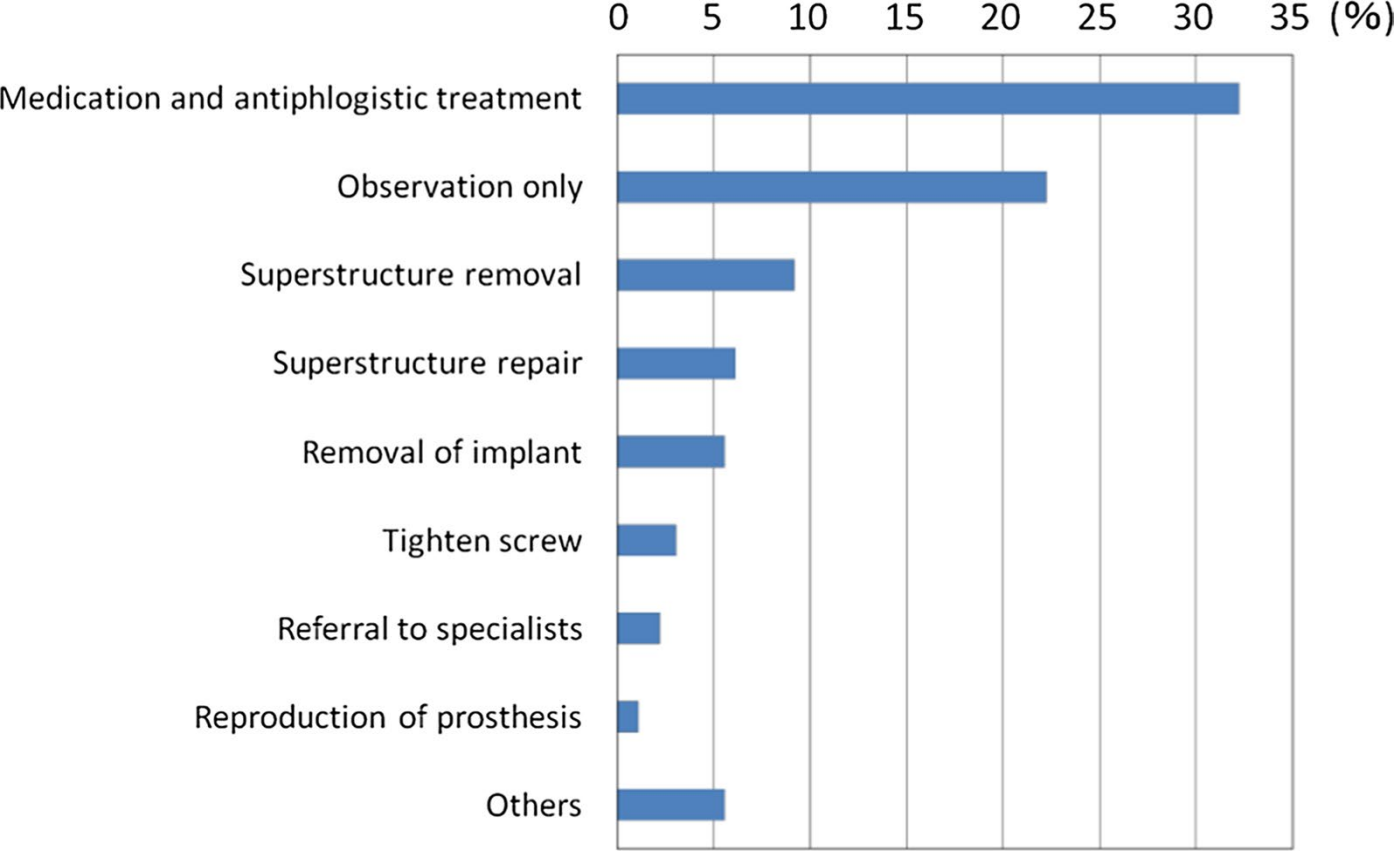
Countermeasures for implant problems taken in the past. Medication and antiphlogistic treatment were most frequently adopted, followed by observation

**Fig. 5:**
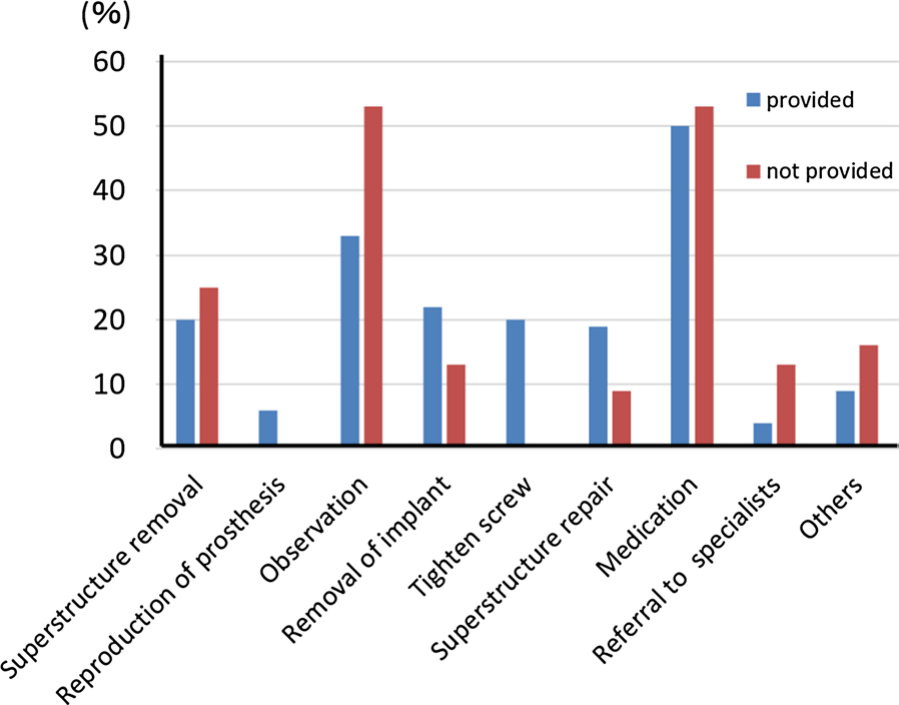
Provision of implant treatment and countermeasures for problems. Many of the dentists who did not provide implant treatment opted for observation or medication while those who provided implant treatment also implemented removal, retightening, repair and so forth

1. Actual state of implants and daily implant care in patients receiving visiting treatment
  The types of implants were, in order of larger numbers, root form type at 87.2% (314 patients), blade type at 9.4% (34 patients), subperiosteal implant type at 4.4% (16 patients) and others at 0.6% (2 patients) (Fig. 1).
  The 96 dentists mentioned above responded that the types of daily implant care provided by dentists, facility staff and so forth in the past 12 months included: brushing with a toothbrush only (49.0%, 47 dentists), combination of a toothbrush and an auxiliary tool (88.5%, 85 dentists), gum massage (39.6%, 38 dentists), salivary gland massage (21.9%, 21 dentists), moisturizing (33.3%, 32 dentists), cleaning with gauze (12.5%, 12 dentists), dedicated care (10.4%, 10 dentists), mouthwash (2.1%, 2 dentists) and others (7.3%, 7 dentists) (Fig. 2).
2. Actual state of implant problems
  Of the 360 patients revealed by the questionnaire, the types and numbers of implant troubles that were encountered in the past were as follows: difficulty of cleaning (45%, 170 patients), peri-implantitis (39%, 139 patients) and fracture of facing material (16%, 59 patients) (Fig. 3).
3. Actual state of countermeasures for implant problems
  The actions that were taken against implant troubles in the past for the 360 patients were as follows: medication/anti-inflammatory measures (32.2%, 116 patients), observation (22.2%, 80 patients), superstructure removal (9.2%, 33 patients), superstructure repair (6.1%, 22 patients), removal of implant (5.6%, 20 patients), tighten screw (3.1%, 11 patients), and referral to specialists (2.2%, 8 patients) (Fig. 4). When these cases are classified by dentists who provided implant treatments and those who did not, the dentists who provided implant treatments conducted the following actions: superstructure removal (22%, 12 patients), tighten screw (20%, 11 patients), and superstructure repair (19%, 10 patients), whereas the dentists who did not often resorted to observation (53%, 17 patients) (p < 0.01) and medication (53%, 17 patients) (Fig. 5).

## Discussion

Our previous report [4] revealed the state of implant treatment provision, state of visiting dental treatment, trends in patients after implant treatment, actual state of implants in visiting dental treatment, and actual state of implant card utilization as well as awareness of it, and indicated that it was essential to expand the provision of continuous care after implant treatment and that popularization of the implant cards under a unified standard was necessary to achieve this.

In this report, we narrowed down the survey respondents to dentists who provided visiting dental treatment, and analyzed the actual state of implant care and problems encountered as well as the countermeasures in order to elucidate the actual state of implants in elderly people requiring nursing care.

1. Actual conditions of implants in visiting care patients and actual conditions of daily care
  While most of the implants encountered (87.2%) were root forms, certain quantities of the blade type and subperiosteal types were also present. Adoption of blade implants started decreasing around 1985 [5], and the use of subperiosteal implants is assumed to have also declined [6]. However, they are still present in some patients, and thus it is considered that education on these systems is still necessary.
2. Actual state of implant problems
  Since many cases involved difficulty in cleaning or peri-implantitis according to the types and numbers of implant troubles that were encountered in the past, we found that there were many troubles related to oral cavity cleaning. It is therefore important to ensure professional care and management. It is also necessary for the dentists to install implant prostheses while taking into consideration the cleaning properties and modifiability of the prosthetic devices.
  We consider it favorable that toothbrush + auxiliary tools (such as interdental brushes) accounted for a majority of daily implant care. However, this study was not able to clarify who provides this care and how, or whether such care is properly implemented. Implant treatment itself has achieved sufficient success rates even in elderly people [7] and people with disabilities [8], as long as the implants are properly managed. It may be difficult for elderly people who require nursing care and who cannot visit a dental clinic to continue self-care or professional care [9]. In their report on three case examples, Visser et al. [10] stated that it was important to ask, “Is the patient supported by a well-functioning oral (self) care assisting network? Is it possible for the patient to regularly see an oral health care professional and is oral health care easily accessible in case of an emergency?” Due to the fact that the rate of people who were incapable of self-care was quite high at 56% [4], it seems that professional care and management are more important, even though there is also an issue of manpower [11–13]. We await the results of more detailed fact-finding studies on oral care in the future.
3. Actual state of countermeasures for implant problems
  Since we found differences in actions taken against implant troubles by dentists who provide visiting dental care depending on their experience and knowledge in implant treatment, it is desirable to expand pre-graduate education on implants, the system for introduction to implant experts, and so forth.
  The fact that many of the dentists who did not provide implant treatment opted for observation or medication while those who provided implant treatment also implemented removal of the upper structure, retightening of screws, repair and so forth suggests that those who did not provide implant treatment found it difficult to take appropriate measures due to insufficient knowledge or skills related to implants. Even though student education on implants has become more substantial in recent years [14], further coverage is desired, including oral care for patients living in a nursing home including elderly people and patients receiving home treatment, comprehension and management of systemic conditions, coordination with other occupations and so forth. In addition, since it is not practical to presume that all dentists providing visiting dental treatment would be capable of sufficient measures regarding implants, coordination with implant specialists should also be examined.
  In addition, the fact that there is insufficient evidence for actions against implant troubles in visiting dental care is a problem. While position papers by experts [15, 16] are beginning to be published, the accumulation of evidence and establishment of guidelines are also necessary. To do this, we need to conduct more surveys on actual conditions in visiting dental care, in order to determine the relationship between individual patients and level of trouble, such as the degree of autonomy of the patient and the level of peri-implantitis [4]. It is also necessary to examine the situation that the handling of implant troubles is not covered by health insurance, which is unique to Japan. Efforts to achieve public consensus will be necessary while also taking into account the increase in medical expenses.

## Conclusion

While field surveys will be required in the future since the present survey was done by questionnaire, we were able to clarify the following points:

1. The most frequent implant troubles encountered by dentists were difficulty of cleaning and peri-implantitis.
2. While many of the dentists who did not provide implant treatments resorted to observation or medication, those who provided implant treatments also removal of implant, repair and so forth.
3. Dentists who provide visiting dental care need to either acquire knowledge and skills in implant treatment or consider coordinating with implant experts.

## Data Availability

The data of this study is not permitted to be widely disclosed by the Ethics Committee of the Society, so we will refrain from disclosing here.
